# Observations on Burrow Use and Reproduction in the Annamite Striped Rabbit (*Nesolagus timminsi*) in Vietnam

**DOI:** 10.1002/ece3.73680

**Published:** 2026-06-07

**Authors:** Linh Van Nguyen, Minh Van Vo, Quy Tan Le, An Nguyen, Andreas Wilting, Kathleen Roellig, Klaus Hackländer, Robyn Hudson, Andrew Tilker

**Affiliations:** ^1^ Southern Institute of Ecology Ho Chi Minh City Vietnam; ^2^ Faculty of Biology, Agriculture and Environment University of Education – The University of Danang Da Nang City Vietnam; ^3^ Institute of Advanced Technology Vietnam Academy of Science and Technology Ho Chi Minh City Vietnam; ^4^ Leibniz Institute for Zoo and Wildlife Research Berlin Germany; ^5^ Department of Ecosystem Management, Climate and Biodiversity BOKU University, Institute of Wildlife Biology and Game Management Vienna Austria; ^6^ Instituto de Investigaciones Biomédicas Universidad Nacional Autónoma de México Mexico City Mexico; ^7^ Re:wild Austin Texas USA

**Keywords:** Annamite striped rabbit, burrow, camera trap, *Nesolagus timminsi*, reproduction, Vietnam

## Abstract

The Annamite striped rabbit ( *Nesolagus timminsi* ) is a forest‐dwelling lagomorph endemic to the Annamite Mountains of Vietnam and Laos. Herein, we report the first detailed observations of its burrowing and reproductive behavior from information gathered via camera traps. From May to October 2024, six camera traps were deployed at suspected burrow sites identified through semi‐structured interviews and field surveys. The active burrows were recorded at multiple sites within broadleaf wet evergreen forests between 359 m and 775 m asl. The camera traps were active for 864 camera trap nights, recording 1293 videos of the species. The videos showed first observations of the Annamite striped rabbit performing a sequence of burrow opening, closing and concealment, including placing compacted soil and small rocks over the entrance. Furthermore, a single behavioral event lasting 190 min was recorded and interpreted as being suggestive of parturition. Forty‐six days after this event, the female was recorded leaving the burrow accompanied by two juveniles. These findings represent the first documentation of such behavior in the species. Our results provide new behavioral information on this little known and endangered animal.

## Graphical abstract

We present the first detailed observations of burrow use and reproductive behavior in the endangered Annamite striped rabbit ( *Nesolagus timminsi* ) based on camera‐trap video data from central Vietnam. The study documents a complete sequence of burrow opening, closing, and concealment, including the novel use of small rocks to cover the nursery burrow entrance, and reports a behavioral event consistent with parturition followed by the emergence of two juveniles. These findings provide critical new insights into the species' reproductive ecology and have direct implications for conservation and ex‐situ management.

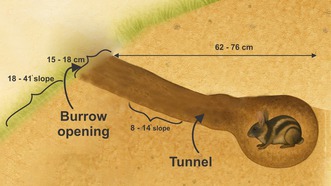

## 1 Introduction

The Annamite striped rabbit ( *Nesolagus timminsi* ) is a rare and elusive leporid endemic to the Annamite Mountains of Vietnam and Laos (Dang et al. 2001; Tilker et al. 2019). The species was first described in 1996 based on specimens from a market in Laos (Surridge et al. 1999), and subsequent surveys have indicated that it is found in wet evergreen forests across the wider Annamites region (Tilker et al. 2019). The species is currently listed as Endangered on the IUCN Red List as a result of population decline caused by widespread snaring across its range (Tilker et al. 2019).

Recent studies have expanded our knowledge of the species' distribution and the main factors influencing its occurrence (Tilker et al. 2019; Nguyen et al. 2023), yet critical aspects of its ecology remain largely unexplored, including its burrow construction, breeding cycle, and reproductive behavior (Schai‐Braun and Hackländer 2016; Tilker et al. 2018). A general distinction between hares and rabbits is their reproductive mode. Hares give birth to precocial young that are born fully furred with eyes open (Chapman and Flux 1991). Young rabbits are altricial and, after a relatively short gestation, are born naked with eyes and ears closed (Chapman and Flux 1991). From this information, it is likely that the Annamite striped rabbit gives birth to young that require extensive parental care in secure burrows. In other rabbit species, mothers construct hidden nests or burrows to protect their offspring from predators and to maintain a stable microclimate (Hansell 1984). Burrow use has been mentioned anecdotally for the Annamite striped rabbit (Abramov et al. 2016; Tilker et al. 2019), but there are no comprehensive data on burrow construction or reproductive behavior for the species.

Herein, we present the first documentation of burrow‐associated behaviors and use associated with reproduction and parental care in the Annamite striped rabbit, though these specific acts were not directly observed but based on camera trap video data in central Vietnam. Understanding burrow use and reproductive behavior is essential for informing conservation planning and potential ex situ management of this threatened species. Our findings provide novel insights into the behavior of this little‐known animal.

## 2 Materials and Methods

We conducted field surveys in the Saola Nature Reserve in Quang Nam Province, central Vietnam, from May to October 2024. The protected area covers 15,822 ha of closed‐canopy wet evergreen rainforest with small patches of secondary and degraded forest. Elevation ranges from 423 m to 1446 m above sea level (asl). It has a tropical monsoon climate with an average annual rainfall of approximately 2500 mm (CRD 2017). Previous camera trap surveys have shown that the site harbors a population of the Annamite striped rabbit that may be regionally important for the long‐term conservation of the species (Tilker et al. 2020).

To identify potential survey sites, we conducted semi‐structured interviews with nine local rangers who had extensive experience with the forest area. These rangers subsequently referred us to 12 local villagers with local ecological knowledge of the forest and, specifically, the Annamite striped rabbit. All interviewees were asked to describe the physical characteristics, behaviors of species, and the specific forest habitats where rabbits and their burrows were most frequently encountered. Initial surveys were then conducted based on this information, leading to the identification of 17 potential burrows. Potential burrows were identified based on three criteria: (1) a single entrance showing signs of excavation but deliberately sealed with compacted soil, clearly distinct from the open burrows of sympatric rodent species; (2) location on sloping terrain, typically on hillsides and often near small forest streams; and (3) presence of fresh footprints around the entrance consistent with the Annamite striped rabbit. At each burrow, we measured entrance diameter, estimated tunnel length, and documented the presence of nest material. For the empty burrows, tunnel length was measured by extending a calibrated flexible rod to the end of the tunnel. For the burrows inhabited by striped rabbits, tunnel length and nest chamber size were not measured to minimize disturbance.

Additionally, to maintain the structural integrity of active nests and minimize disturbance of the breeding rabbit and its offspring, we did not excavate the burrow or remove nesting materials for detailed botanical and soil analysis. This minimally invasive approach was adopted to prevent nest abandonment, a known risk in sensitive lagomorphs when faced with anthropogenic micro‐habitat alteration (Gillingham and Parker 2008). We used a compass to measure the orientation of each burrow, recorded the elevation using a Garmin GPSMAP 64s device (Garmin Ltd., Schaffhausen, Switzerland), and the slope of the surrounding terrain using a Suunto Tandem/PM‐5 clinometer (Suunto Oy, Vantaa, Finland). We then deployed a camera trap (Spartan GoCam Lumen Dual Flash Scouting Camera, Spartan Camera, Duluth, GA, USA; or Bushnell Core DS 30MP No Glow, model 119977C, Bushnell Outdoor Products, Overland Park, KS, USA) facing the entrance of each burrow. Prior to deployment, we extensively searched for possible additional entrances within a 5 m radius of the burrows. None were found, which indicated that all burrows likely had only a single entrance. We classified burrows as active only if visits by rabbits were confirmed by the videos from camera traps. We mounted camera traps 20–30 cm above the ground on tree trunks and cleared away surrounding vegetation to ensure an unobstructed field of view (Caravaggi et al. 2017). The cameras were programmed to take a 15–30 s video per trigger, with a 10 s delay between triggers. Trigger sensitivity was set to high to maximize detection probability. Cameras were operated continuously, 24 h per day, and stayed in the forest for at least 60 days.

To ensure consistency and replicability in the processing of behavioral data, we developed an ethogram following the guidelines of Bateson and Martin (2021). We reviewed all videos and recorded burrow visits, defined as discrete periods beginning when a rabbit arrived at the burrow entrance and ending upon its departure. We then classified activities within each visit into six predefined categories, including burrow opening, closing, parturition, offspring emergence, vigilance, and resting (Table 1). These definitions provided consistent temporal boundaries for analysis and improved the accuracy of both frequency and duration measurements of the burrow‐associated behaviors. Using these data, we inferred several aspects of the species' reproductive ecology, including temporal aspects and the potential function of burrow use and maternal behaviors in offspring protection.

**TABLE 1:** Definition of Annamite striped rabbit ( *Nesolagus timminsi* ) behaviors observed at a nursery burrow.

Behavior category	Description
Burrow opening	Using forelimbs or snout to clear soil from or to enlarge the burrow entrance
Burrow closing and concealment	Covering the burrow entrance with soil, stones, leaves, or nearby vegetation
Parturition behavior	Facing outward at the burrow entrance, hindquarters inside, with rapid breathing indicative of parturition
Offspring emergence	Juveniles emerging from and leaving the nursery burrow
Vigilance posture	Remaining alert at the entrance, with frequent head and ear movements presumably to scan for threats
Resting	Sitting quietly near the burrow entrance, appearing to rest

## 3 Results

### 3.1 Burrow Characteristics

Of the 17 potential burrows, six were actively used by Annamite striped rabbits as confirmed by camera trap videos. These six active burrows were found across an elevation range of 359 to 775 m asl, located in closed‐canopy wet evergreen forest, characterized by dense understory vegetation. One burrow was located immediately adjacent to a major road, with the entrance just 2.5 m from the roadside. The average slope of the surrounding terrain at burrow sites was 29.5° (18°–41°). Of the six active burrows, four entrances faced southeast and two faced east.

**FIGURE 1:**
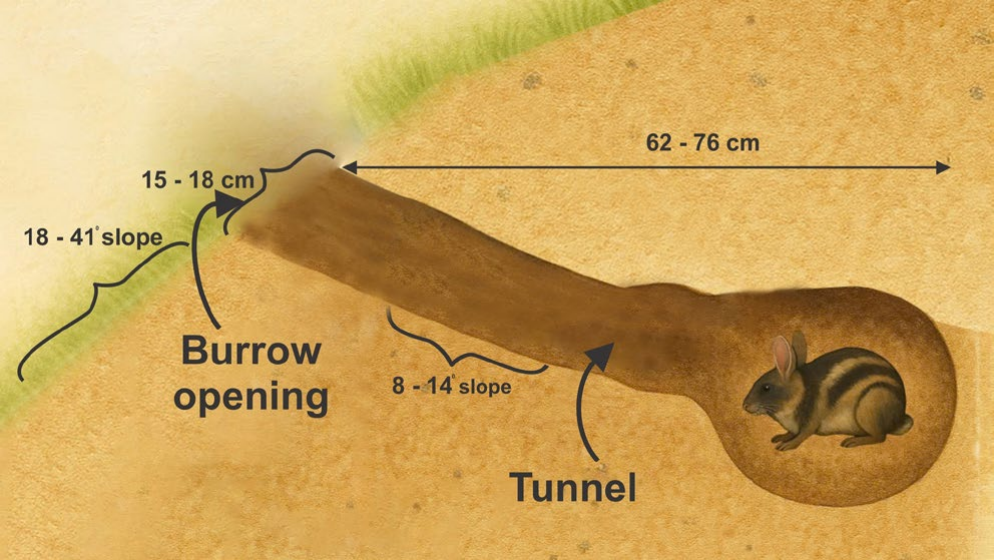
Schematic diagram of an Annamite striped rabbit ( *Nesolagus timminsi* ) burrow in the Quang Nam Saola Nature Reserve, Vietnam.

Burrow entrances measured were 15–18 cm in diameter. Internally, the burrows had a simple structure consisting of a single straight tunnel 62–76 cm in length. The internal slope of the burrows was relatively shallow, ranging from 8° to 14° with a mean gradient of 11**°** (Figure 1). All active burrows contained nesting material, primarily composed of dry leaves and grass.

### 3.2 Burrow Activity

Across 864 camera trap nights, the six camera traps placed at the active burrows recorded 1293 videos, yielding 91 burrow visits. These events typically included a complete sequence consisting of opening the burrow entrance, entering the burrow, closing it, and entrance “concealment”, with repetition of the same activities on consecutive days. Burrow opening to closing lasted an average of 45 min (range: 29–61 min) per event. Entrance concealment was observed for all 91 visits across all six burrows.

**FIGURE 2:**
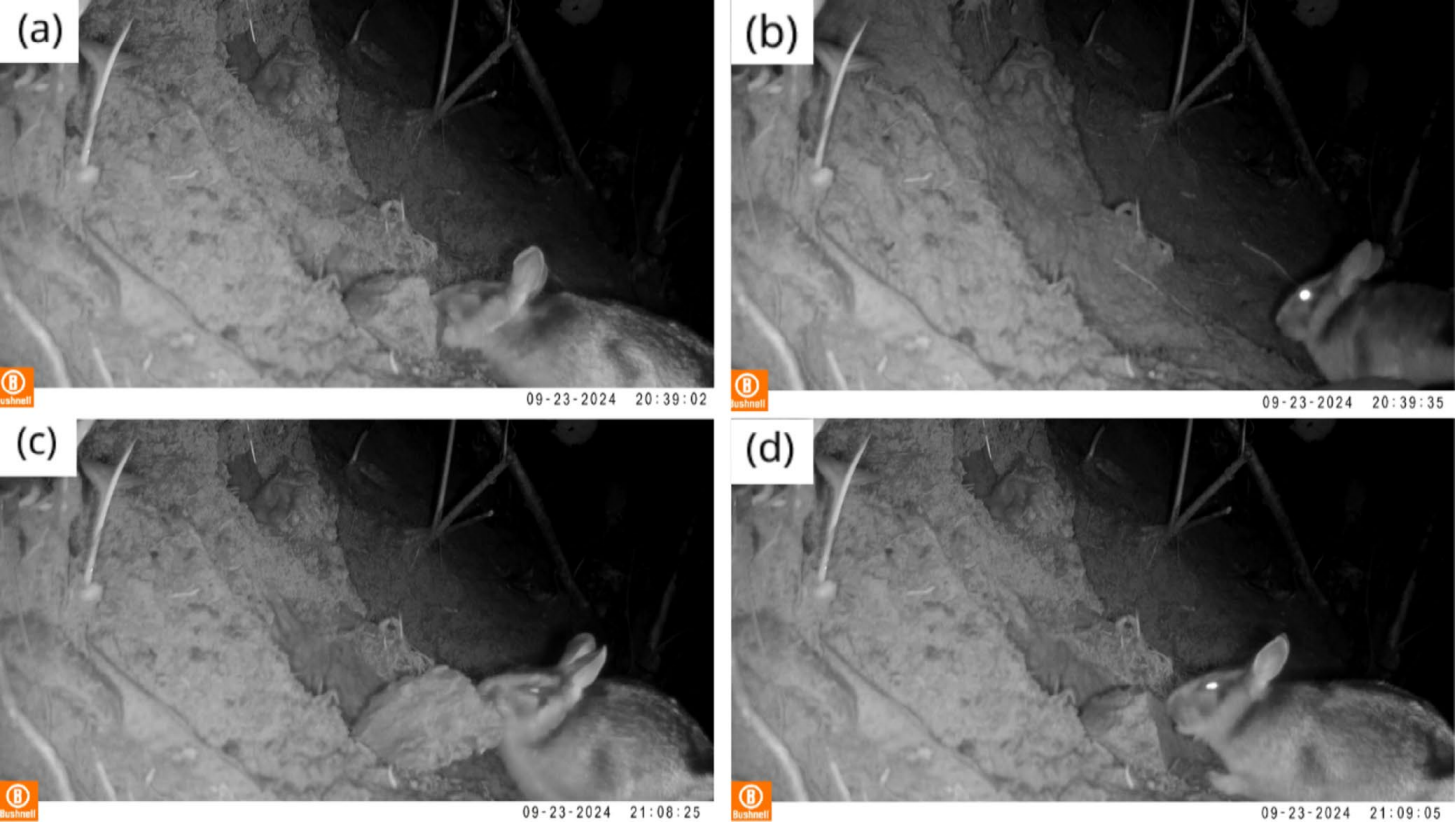
Screenshots illustrating burrow opening and closing activities of an Annamite striped rabbit ( *Nesolagus timminsi* ) recorded at Saola Nature Reserve, Quang Nam Province, Vietnam. (a, b). The rabbit is seen opening the burrow by using its mouth and front legs to loosen and move rocks or debris sealing the entrance (at 20:39 h). (c, d) Approximately 29 min later (21:08–21:09 h), the rabbit is seen concealing the entrance by using its body and limbs to push or roll small rocks and debris over the entrance.

At each visit, the rabbit initially used its forelimbs to open the burrow, scraping away loose soil from the entrance, followed by closing the burrow using both its fore‐ and hind limbs. On 37 visits (40.6%), rabbits were recorded using their teeth to break apart compacted soil and then using this to close the entrance. At one burrow, we recorded five separate occasions of the rabbit using small rocks to cover the burrow entrance (Figure 2). Based on video observations, the materials used to conceal the burrow entrance were small, irregular stones or pebbles, visually estimated to be approximately 3–5 cm in diameter. We did not conduct physical measurements of these items to avoid human disturbance at the active nest site. All 91 burrow visits (100%) occurred during the nighttime, from 19:52 h to 02:21 h.

### 3.3 Reproductive Activity

**FIGURE 3:**
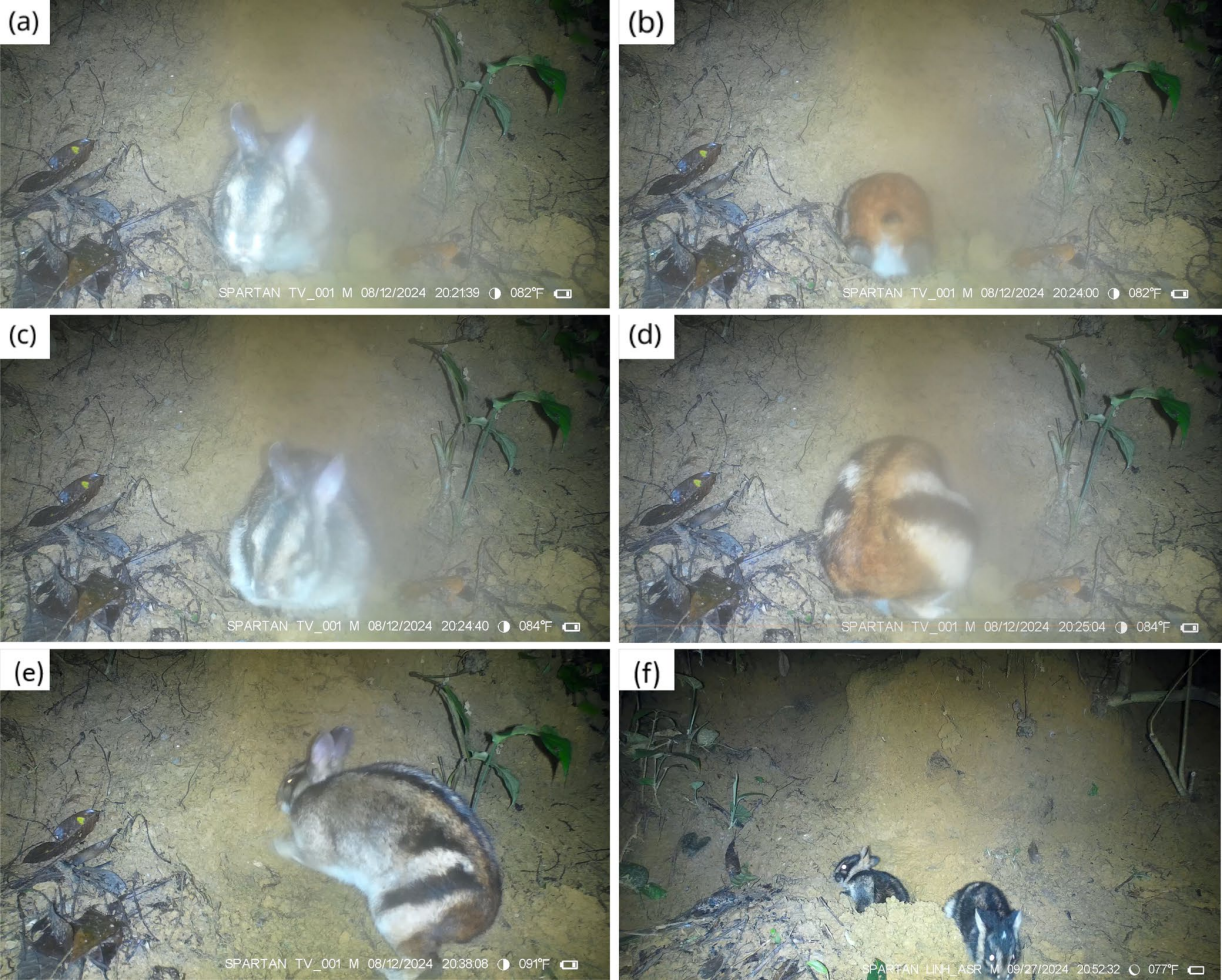
Apparent parturition and emergence of offspring of an Annamite striped rabbit ( *Nesolagus timminsi* ) recorded at Saola Nature Reserve, Quang Nam Province, Vietnam. (a) Behavior indicating the approach of parturition or parturition itself; (b) entering the burrow; (c) second behavioral sequence again indicating imminent parturition or parturition itself; (d) re‐entering the burrow; (e) closing the burrow; (f) two juveniles leaving the burrow.

We also recorded one event that was suggestive of reproductive activity. On August 12th, 2024, a presumably female rabbit, based on behavioral observation, including the presence of young rabbits in the burrow, was observed performing a complete burrow‐attendance cycle, including opening the entrance, entering, exiting, and closing the burrow (Figure 3). This event began at 20:15 and lasted 190 min, longer than any other recorded visit. During this time, the female displayed two unusual behaviors not observed during other visits: (1) she repeatedly positioned herself at the burrow entrance with her head facing outward and tail inside, and (2) she repeatedly paused in this position while exhibiting heavy breathing before then re‐entering the burrow. Given the synchronized occurrence and persistence of these two behaviors, we hypothesize this sequence likely represented pre‐partum behavior or parturition itself (Hamada and Mizuta 2020). No similar behaviors were documented at other burrows during the study.

During the subsequent 46‐day monitoring period, the adult individual was repeatedly recorded entering and exiting the burrow. On Day 46 following the previous observations, the adult was recorded reopening and further excavating the burrow entrance before entering; shortly thereafter, the adult and two juveniles emerged from the burrow together. Prior to departing, the adult sealed the burrow entrance. No further activity was recorded in subsequent footage, and the burrow remained completely sealed when the team retrieved the camera trap 25 days after this event.

## 4 Discussion

To our knowledge, this study provides the first documentation of burrow opening, closing and covering the entrance of a nursery burrow associated with reproduction and care of young in the Annamite striped rabbit, and more broadly, within the genus *Nesolagus*. Our camera trap data suggest that this species not only uses and modifies its nursery burrows, but also employs a concealment strategy involving soil, vegetation, and small rocks. It is likely that the burrow concealment behavior that we observed reflects an evolved adaptation to protect the altricial offspring from predation. Construction of a nursery burrow and entrance‐concealing behavior have been documented in the pygmy rabbit ( *Brachylagus idahoensis* ; Rachlow et al. 2005; Nielsen and Berkman 2018), Mexican cottontail ( *Sylvilagus cunicularius* ; Rodríguez‐Martínez et al. 2014; Lorenzo et al. 2018), and riverine rabbit ( *Bunolagus monticularis* ; Bragg et al. 2018). The Amami rabbit ( *Pentalagus furnessi* ) has also been reported to rapidly cover the entrance to its nursery burrow using soil, leaves, and grass (Fumio and Cervantes 2005; Hamada and Mizuta 2020), suggesting this to be a highly conserved pattern of maternal behavior in diverse rabbit species across widely separated geographic regions. However, to our knowledge, no other rabbit has been recorded using small rocks to cover the burrow entrance. This specialized behavior, observed at a burrow with reproductive activity, could indicate that Annamite striped rabbits use small rocks to create a particularly robust barrier to protect vulnerable young. We observed a consistent southeast–east orientation of active burrows. While our small sample size precludes drawing definitive ecological conclusions, this pattern warrants further investigation to determine if it reflects environmental preferences, such as microclimate optimization, or is simply constrained by local topographical features. Future studies with larger sample sizes and localized microclimatic data will be necessary to test these possibilities.

The observation of reproductive behavior in this study is the first record for the species, establishing a basis for continued research on this behavior in the wild. However, data on the timing of reproduction show a notable discrepancy with the single other study that mentions reproductive behavior in the species. Abramov et al. (2016) recorded young at Pu Mat National Park (located more than 400 km north of our study area) in May 2016, whereas in our study the presumed parturition event occurred in August, with juveniles first recorded emerging from the burrow in the current study in September. This difference may be due to the influence of geographical location on the timing of reproduction, or the species may be capable of multiple breeding events per year. Further field research is required to understand the species' breeding cycle and reproductive parameters.

We note that this study is limited by a small sample size and a relatively short monitoring period, which may not fully represent temporal or spatial variation in reproductive behavior. Furthermore, our field efforts were primarily focused on documenting the physical architecture and dimensions of nursery burrows, such as entrance diameter and tunnel length. Consequently, we did not collect quantitative micro‐habitat data regarding the composition of surrounding understory vegetation. Future studies should expand monitoring across multiple sites and extend observation duration to encompass seasonal and interannual dynamics, and integrate detailed botanical surveys to better understand the factors influencing burrow site selection.

Our findings provide information that may help guide ex‐situ conservation programs for the species, especially relating to reproduction and maternal care. However, information on parameters relating specifically to husbandry will require further targeted research. Because the population decline of the Annamite striped rabbit through snaring shows little sign of abating, conservationists have called for the establishment of ex‐situ insurance populations of the species (Tilker et al. 2019). Understanding the Annamite striped rabbit's behavioral ecology, particularly its behavior relating to its breeding burrow, may help to successfully keep and breed the species in conservation breeding facilities, thereby contributing to its long‐term survival. While our study does not provide a comprehensive captive husbandry protocol, it provides information on parameters critical for the design of naturalistic enclosures. Specifically, our data on burrow length (62–76 cm), tunnel structure, and the presence of nesting material (dry leaves and grass) suggest that ex‐situ facilities should prioritize enclosure designs that allow for natural digging and burrowing behaviors. Replicating natural conditions in ex‐situ facilities will help maximize opportunities for conservation breeding of the species in the future, as has been recommended by conservationists (Tilker et al. 2019). Currently, a conservation breeding center has been established at Bach Ma National Park in central Vietnam, immediately adjacent to the Saola Nature Reserve, which includes in its strategy the establishment of an ex‐situ population of the Annamite striped rabbit (MARD 2023). However, before future reintroductions can take place, it will be important that sites are secure, with demonstrable reductions in snaring pressure. Reducing threats to levels that allow reintroductions will likely be a long‐term endeavor, but with appropriate resources and political support, it is possible.

## Author Contributions

**Linh Van Nguyen:** conceptualization (equal), data curation (equal), formal analysis (equal), investigation (lead), methodology (equal), project administration (lead), resources (equal), visualization (equal), writing – original draft (equal), writing – review and editing (equal). **Minh Van Vo:** conceptualization (equal), methodology (equal), project administration (equal), supervision (equal), writing – original draft (equal). **Quy Tan Le:** investigation (equal), methodology (equal), writing – original draft (equal), writing – review and editing (equal). **An Nguyen:** investigation (equal), methodology (equal), writing – original draft (equal), writing – review and editing (equal). **Andreas Wilting:** investigation (equal), methodology (equal), writing – original draft (equal), writing – review and editing (equal). **Kathleen Roellig:** investigation (equal), methodology (equal), writing – original draft (equal), writing – review and editing (equal). **Klaus Hackländer:** conceptualization (equal), writing – review and editing (equal). **Robyn Hudson:** conceptualization (equal), writing – review and editing (equal). **Andrew Tilker:** conceptualization (equal), formal analysis (equal), methodology (equal), visualization (equal), writing – review and editing (equal).

## Funding

This research was funded and supported by the Southern Institute of Ecology; the Institute of Advanced Technology, Vietnam Academy of Science and Technology; and the Leibniz Institute for Zoo and Wildlife Research.

## Ethics Statement

This study was conducted under the academic framework of the University of Education, Danang (UED), specifically authorized by Letter of Introduction No. 75/GGT‐DHSP dated April 30, 2024. The research proposal was formally reviewed and approved by the management of the Quang Nam Saola Nature Reserve, granting full access for fieldwork.

## Conflicts of Interest

The authors declare no conflicts of interest.

## Data Availability

The data that support the findings of this study are openly available in Zenodo at https://doi.org/10.5281/zenodo.15274968.
